# Reduced graphene oxide induces transient blood–brain barrier opening: an in vivo study

**DOI:** 10.1186/s12951-015-0143-z

**Published:** 2015-10-30

**Authors:** Monique Culturato Padilha Mendonça, Edilene Siqueira Soares, Marcelo Bispo de Jesus, Helder José Ceragioli, Mônica Siqueira Ferreira, Rodrigo Ramos Catharino, Maria Alice da Cruz-Höfling

**Affiliations:** Department of Pharmacology, Faculty of Medical Sciences, State University of Campinas, Campinas, SP Brazil; Department of Biochemistry and Tissue Biology, Institute of Biology, State University of Campinas, Campinas, SP Brazil; Department of Semiconductors, Instruments and Photonics, Faculty of Electrical and Computer Engineering, State University of Campinas, Campinas, SP Brazil; Department of Medicine and Experimental Surgery, Faculty of Medical Sciences, State University of Campinas, Campinas, SP Brazil

**Keywords:** Blood–brain barrier, Paracellular pathway, Nanomaterials, MALDI-MSI

## Abstract

**Background:**

The blood–brain barrier (BBB) is a complex physical and functional barrier protecting the central nervous system from physical and chemical insults. Nevertheless, it also constitutes a barrier against therapeutics for treating neurological disorders. In this context, nanomaterial-based therapy provides a potential alternative for overcoming this problem. Graphene family has attracted significant interest in nanomedicine because their unique physicochemical properties make them amenable to applications in drug/gene delivery and neural interface.

**Results:**

In this study, reduced graphene oxide (rGO) systemically-injected was found mainly located in the thalamus and hippocampus of rats. The entry of rGO involved a transitory decrease in the BBB paracellular tightness, as demonstrated at anatomical (Evans blue dye infusion), subcellular (transmission electron microscopy) and molecular (junctional protein expression) levels. Additionally, we examined the usefulness of matrix-assisted laser desorption/ionization (MALDI) mass spectrometry imaging (MSI) as a new imaging method for detecting the temporal distribution of nanomaterials throughout the brain.

**Conclusions:**

rGO was able to be detected and monitored in the brain over time provided by a novel application for MALDI-MSI and could be a useful tool for treating a variety of brain disorders that are normally unresponsive to conventional treatment because of BBB impermeability.

## Background

The blood–brain barrier (BBB) has an intricate physical and molecular structure that provides a proper microenvironment for neuronal cell activity. The physical structure consists of a fenestration-free continuous endothelium and a surrounding basement membrane covered by a sheath of distal end-feet of astrocytes processes and pericytes. The molecular structure consists of a collection of membrane receptors and highly selective carriers that regulate the bi-directional trans-endothelial movement of molecules across the blood–brain interface. The inter-endothelial trafficking of molecules is prevented by an elaborate, closely applied junctional contact that is provided by tight junctional and adhesion transmembrane proteins, some of which are anchored to the cell cytoskeleton. Such an intricate arrangement results in brain endothelial lining that has a very high electrical resistance compared to peripheral vascular endothelium [1]. The BBB is a highly dynamic structure that responds to minimal changes in the circulating blood and brain microenvironment through self-adjusting mechanisms that serve to protect neuronal activity. However, the complexity of the physical and molecular arrangement of the BBB precludes the accessibility of many drugs to the central nervous system. As a consequence, various neurological disorders remain untreatable and this can be an important cause of precocious death.

The emerging interdisciplinary field of nanotechnology could provide a means of overcoming the restrictive nature of the BBB with regard to drug entry into the brain. There has been considerable effort in searching for nanoscale-dimensioned materials amenable to crossing the BBB and delivering drugs to specific sites of injury [2]. Among these nanomaterials, graphene and derivatives, such as reduced graphene oxide (rGO), have attracted significant interest; their free π electrons and extremely high surface area allow interaction with a variety of biomolecules, making them particularly amenable to applications in tissue engineering, molecular imaging, drug/gene delivery [3] and neural interfaces [4, 5].

An important aspect in the development of drug nanocarriers is that of ascertaining their fate in the brain and determining the amount and the way they reached the target. A number of in vivo techniques have been developed to detect and measure the uptake of drug nanocarriers in the brain. Usually, scanning and transmission electron microscopy (TEM), fluorescence microscopy, confocal laser microscopy and pharmacokinetic and biodistribution studies have been used for this purpose [6]. However, these techniques present substantial technical challenges and monitoring the spatial distribution of nanomaterials over time is not always easy or fast.

In recent years, matrix-assisted laser desorption/ionization (MALDI) mass spectrometry imaging (MSI) has emerged as a powerful methodology to investigate the spatial distribution of specific biomolecules in tissue sections [7]. MALDI-MSI has been used to visualize atoms and small molecules at spatial resolutions below one micron [8, 9], which is close to that of nanoparticles with at least one dimension ≤100 nm [10]. The present study was undertaken to test the hypothesis that MALDI-MSI could be used to detect and follow the spatial density of rGO in the brain. For comparative purposes, we also assessed the detection of rGO by confocal laser microcopy.

Another highly significant aspect to consider when studying drug transport and delivery to the brain via nanomaterials relates to the integrity of the BBB throughout treatment. To address this matter, we used three approaches to examine BBB integrity, namely: (a) gross anatomical observation based on the peripheral infusion of Evans blue dye, (b) sub-cellular evaluation by TEM in conjunction with the extracellular tracer lanthanum nitrate to determine whether the tracer escaped into the brain parenchyma, and (c) western blotting (WB) to assess possible cell–cell or cell–matrix disarray in endothelial tight and adhesion junctional proteins and laminin in brain tissue homogenates.

## Results and discussion

### Physicochemical characterization of rGO

The uptake of nanoscale materials from the circulation and their internalization by the brain depends on their physicochemical characteristics, such as morphology, composition, uniformity, size and surface charge [11]. Graphene is composed of a single-atom-thick sheet of sp2-bonded carbon atoms hexagonally arranged in a two-dimensional structure that creates a large surface area on both sides of the planar axis. Graphene family-based nanomaterials include single- or few-layer-graphene, ultrathin graphite, graphene oxide, and rGO [12].

**Fig. 1:**
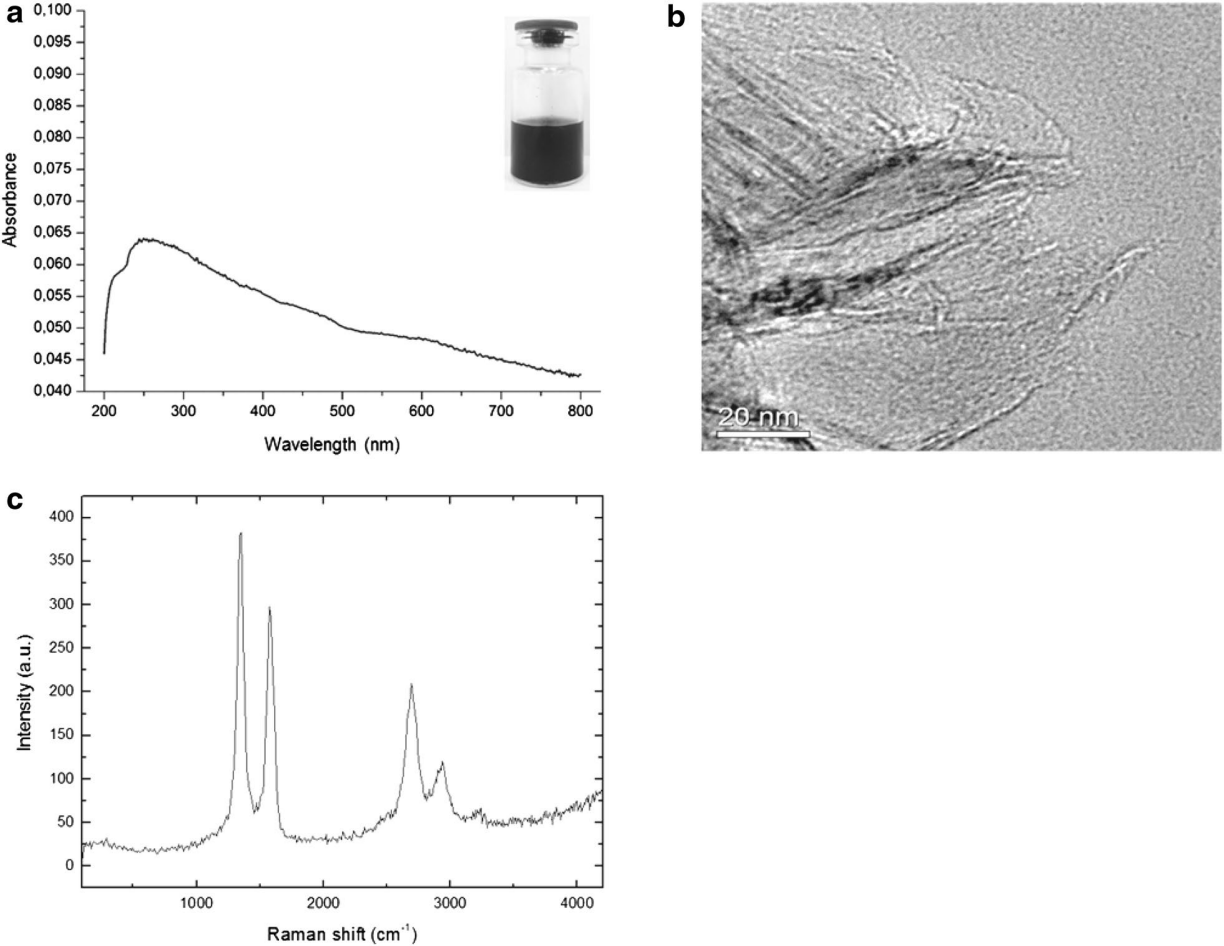
rGO morphology and physicochemical characterization. **a** UV–Visible absorbance spectrum of rGO (70 µg/ml, 265 nm). *Inset* shows the corresponding rGO suspension photograph. **b** HRTEM image showing part of the rGO morphology. **c** Raman spectrum of rGO with the excitation wavelength set to 514.5 nm (Ar ion laser). The C–C sp3 peak of the GO appears at ~1350 cm^−1^ (D-band) and the sp2 peak at ~1598 cm^−1^ (G-band). *a.u.* arbitrary units

rGO was the product of treating graphene oxide under reducing conditions (chemical, thermal, microwave, photo-chemical, photo-thermal or microbial/bacterial) in order to reduce its oxygen content [13]. The reduction degree of the rGO engineered in our laboratory was investigated using UV–Visible spectroscopy. rGO in water results in a black suspension (inset Fig. 1a), which is characteristic of the reduced form of graphene oxide [14]. UV–Visible spectrum (Fig. 1a) exhibited an absorption peak at 265 nm, red-shifted when compared with graphene oxide, which exhibits a π–π* absorption band at 230 nm [15]. The red-shift effect can be explained by a partial restoration of the π network among carbons in rGO, due to removal of the oxygen-containing bonds resulting in electronic conjugation within reduced sheets [16].

rGO was stable in sterile distilled water for over a month without forming agglomerates or changing its physicochemical characteristics. This relatively stable aqueous suspension of rGO can be attributed to the electrostatic repulsion due to the negatively charged sheets (zeta potential of −25 ± 0.18 mV) and the presence of residual oxygen functional groups at defect sites [17]. For the experiments, freshly-suspended rGO was characterized with regard to particle size, zeta potential and polydispersity index (PDI) by dynamic light scattering analysis at pH 7.6 and 25 °C; rGO showed an average diameter of 342 ± 23.5 nm and a PDI of 0.56 ± 0.03. These results indicate that although the rGO was nano-size scale it was polydispersed, as indicated by the PDI value.

The surface morphology and structural parameters of the samples were determined by high resolution transmission electron microscopy (HRTEM) and Raman spectroscopy, respectively. Raman spectroscopy is one of the most powerful techniques for characterizing graphene-based materials [18]. The rGO sheet seen by HRTEM (Fig. 1b) had a relatively large surface area and its morphology resembled a thin curtain. The Raman spectrum of rGO displayed two main bands, D (1350 cm^−1^) and G (1598 cm^−1^) (Fig. 1c). These vibrational band signatures are shared by all sp2-bonded carbon atoms and correspond to the defects or edges (D-band) and to the first order Raman scattering in the E2 g mode (G-band) [19].

### rGO distribution in the brain as determined by MALDI-MSI

MALDI-MSI has previously been used to locate drugs [20, 21] and lipids [22] in rodent brains. In the present study, MALDI-MSI was used to demonstrate the presence, distribution and density of rGO within the brain over time. To map rGO by MALDI-MSI, we used a protocol established for general sample processing that included matrix-assisted laser desorption/ionization, mass analysis and image registration. A detailed step-by-step description and discussion of the MALDI-MSI technique has been published [23, 24]. Although common to all MSI platforms, the general procedure described here requires optimization of the experimental conditions to suit the biological samples in order to obtain the best results.

**Fig. 2:**
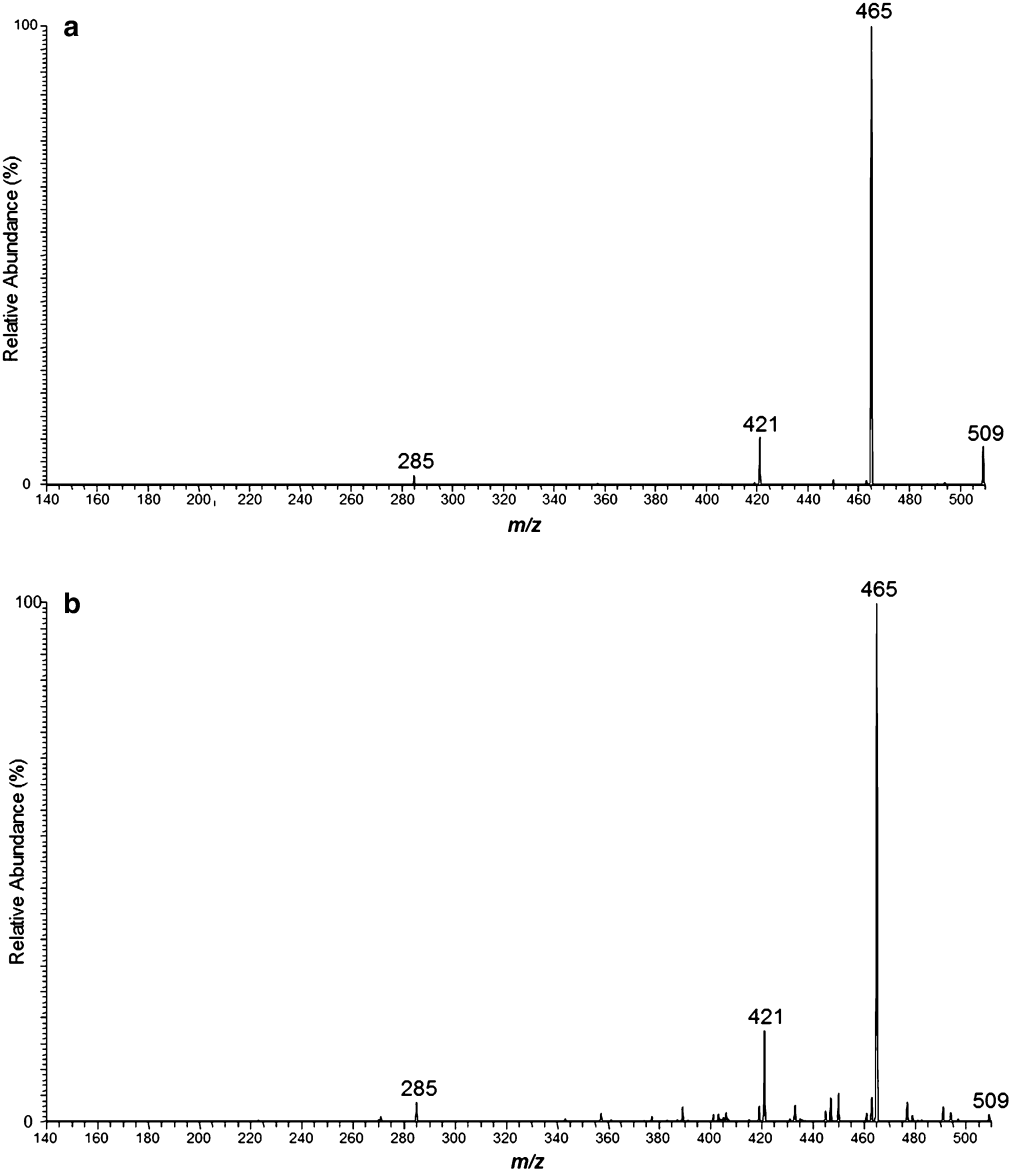
Compiled mass spectra of **a** an aqueous suspension of rGO and **b** hippocampal tissue 1 h after rGO injection in rats. The data were collected in negative ion mode using sinapic acid as the matrix

After adjusting our experimental model with regard to sample preparation and matrix application, the fragmentation pattern of the rGO was confirmed. The laser scanning of the tissue sections revealed four dominant peaks at mass-to-charge (*m*/*z*) ratios of 285, 421, 465 and 509 (Fig. 2). The fragmentation pattern was the same for every rGO administration.

**Fig. 3:**
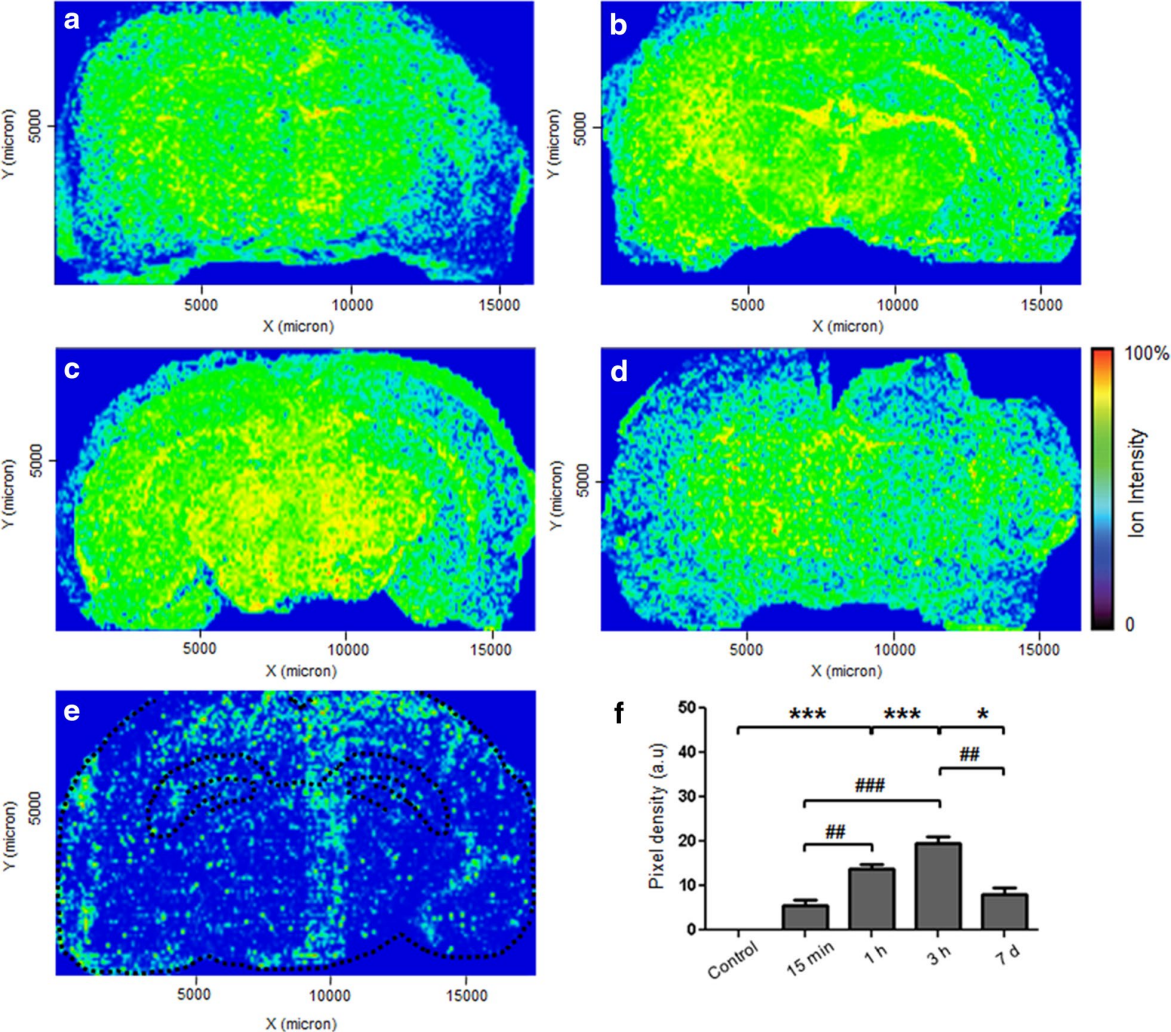
MALDI-MSI images of a coronal section of rat brain tissue and the density of rGO in the brain. The rats received a single dose (7 mg/kg) of rGO via tail vein and were killed at **a** 15 min, **b** 1 h, **c** 3 h and **d** 7 days after administration. **e** Representative distribution of *m*/*z* 465 signal in control animals; the central *black dashed line* delineates the hippocampus boundaries. The *color* scale shows the ion intensity of the rGO (*blue color* for the lowest signal and yellow/red color for highest). The evaluation in arbitrary units (a.u.) of pixels intensity (shown in **f**) was normalized by discounting the matrix background. **f** Quantification of rGO in brain at different times after administration. The *columns* represent the mean ± SEM (n = 3 images/interval; one image per animal). *p < 0.05 and ***p < 0.001 compared to the corresponding control. ^##^p < 0.01 and ^###^p < 0.001 between rGO-treated time-points (one-way ANOVA followed by the Bonferroni post hoc test)

Based on the mass-to-charge ratios of the ions detected, composite images were constructed by mapping the distribution of rGO throughout the rat brain over time (data not shown). The yellow points in Fig. 3 represent the abundance of ions with the molecular mass *m*/*z* 465 located in rat coronal brain sections (Fig. 3a–d).

MALDI-MSI revealed the uptake of rGO and demonstrated their spatial and temporal distribution. At 15 min post-administration, rGO was distributed throughout the brain, with the highest concentration being located mainly in two brain regions, the thalamus and hippocampus. Quantitative analysis of the mean of pixel densities (ion intensity) as a function of the rGO density and measured in the two brain regions revealed a significant progressive increase in rGO content during the first 3 h (Fig. 3f); this increase indicated a continuous movement of rGO from peripheral blood into the brain; 7 days after rGO administration the nanomaterial content was still significantly higher than in the control, and an increase equal in magnitude to that seen 15 min after administration. This reduction in content at 7 days indicated rGO clearance from the brain.

The large size of rGO (342 ± 23.5 nm) was apparently not an obstacle to their entrance into the brain. Very few reports have described the presence of large particles (~200–400 nm) inside the brain [25, 26] and none of them provides a clear explanation of the mechanism by which nanoparticles reach the brain. An understanding of how larger particles enter the brain is of interest because this would allow better efficiency in drug loading, greater dispersion of drugs, and drug release over longer periods of time [27, 28].

### rGO detection by confocal laser microscopy

**Fig. 4:**
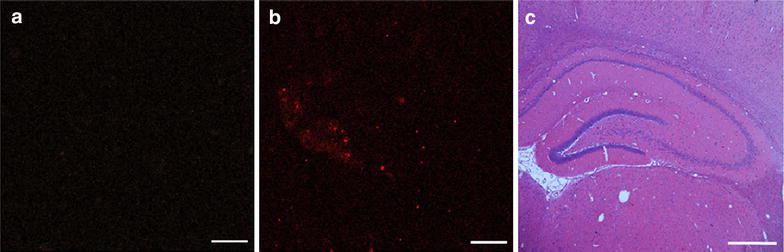
Visualization of rGO by confocal laser microscopy and light microscopy. Representative confocal micrographs of hippocampus at the right cerebral hemisphere from: **a** vehicle-injected (control) rats showing none evidence of nanoparticles and **b** rGO-treated rats (1 h) showing differently-sized *red dots* that indicate the differential accumulation of nanoparticles, mainly in the dentate gyrus. Images were obtained at an excitation wavelength of 365 nm. **c** Bright field image of paraffin-embedded hippocampus (captured from the same region as **a** and **b** panels) from rats administered rGO (1 h): there were no morphologically detectable alterations. Hematoxilyn-eosin. *Scale bars* 20 μm (**a**, **b**) and 200 μm (**c**)

The presence of rGO inside the brain as detected by MALDI images was corroborated by using confocal laser microscopy. After using two-photon excitation with 780-nm laser pulses, rGO emits fluorescence, mostly in the orange-red region of the visible spectrum. The presence of rGO was detected throughout the brain parenchyma, but their density was highest in the thalamus and also with relevant density in hippocampus, with the intensity of fluorescence indicating the spatial abundance. Figure 4 illustrates the presence of fluorescent rGO in the hippocampus, with predominance of rGO clusters in the dentate gyrus.

### Evaluation of BBB integrity

We next examined whether the time-related movement of rGO into the brain of treated rats, as documented by MALDI-MSI and confocal laser microscopy, involved breakdown of the BBB. Figures 3 and 4 gave evidences of the presence of rGO inside the brain parenchyma but did not show the nanoparticles crossing the BBB. As mentioned earlier, the mechanism by which nanoparticles reach the brain is of medical interest [29]. We hypothesized that for rGO to get access into the brain, the BBB had to have been disrupted once the BBB is the fence interposed between the peripheral blood stream (through which the nanoparticles were administered through the tail vein) and the brain. In addition, it would be necessary to determine which transport route was affected. To examine such questions, three-resolution level investigations were implemented.

### Gross anatomical evaluation level

**Fig. 5:**
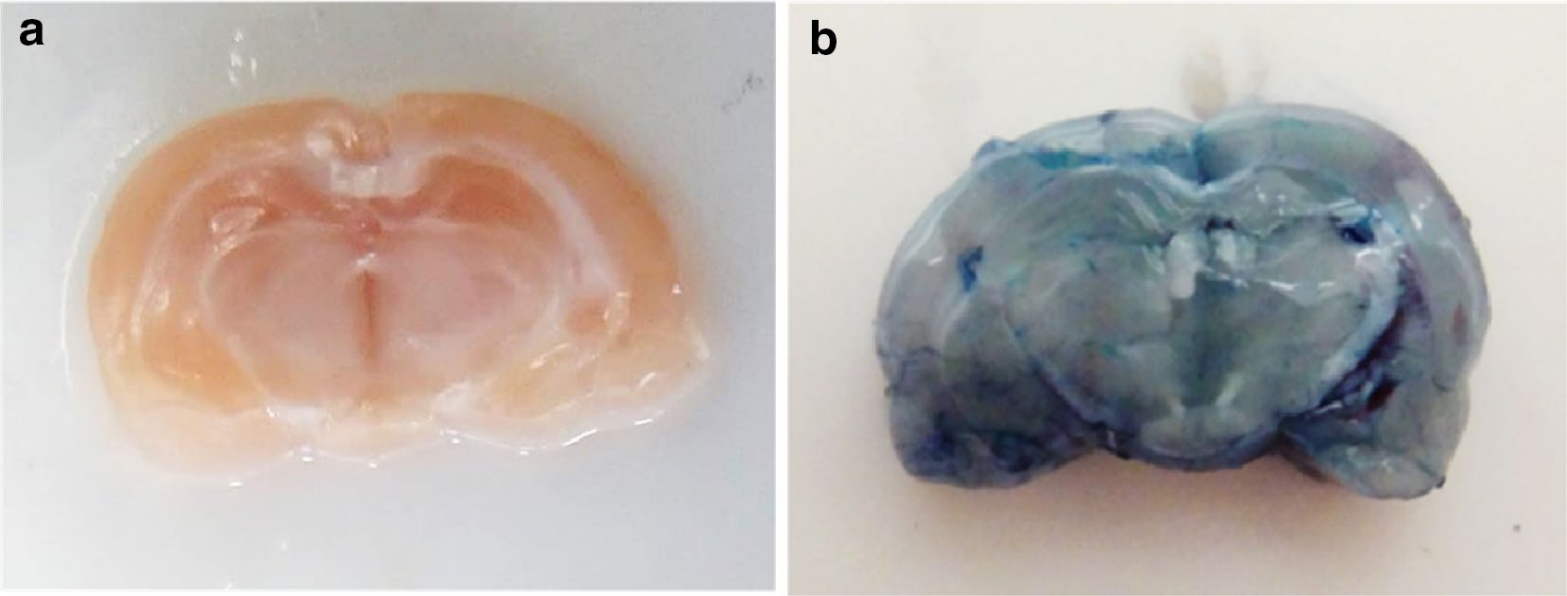
Gross view of a whole brain after infusion with Evans blue dye. **a** Note that none of the dye escaped from the vascular bed in control rat brain, whereas (**b**) dye escaped from the vascular bed in the brain after 3 h of rGO administration, indicating blood–brain barrier leakage

BBB integrity was initially assessed based on gross anatomical observation after peripheral i.v. infusion of the vital dye Evans blue. Since infused Evans blue dye cannot permeate an intact BBB [30], any entry of the dye into and spread within the brain is indicative of a leaky BBB. The systemic infusion of Evans blue in treated control rats did not alter the typical pink color of fresh brain (Fig. 5a). In contrast, in rats administered rGO, the brain was stained a bluish gray color, indicating leakage of the dye from peripheral bloodstream into the brain parenchyma; this staining provided unmistakable evidence of BBB opening (Fig. 5b). This disruptive effect on the BBB was transient and reversible, since dye leakage was observed at 15 min, 1 and 3 h following administration of rGO, but was absent at 7 days. Staining with Evans blue has long been used in different experimental models as a reliable method for assessing BBB integrity and/or leakiness [31, 32].

### Subcellular evaluation level of BBB opening based on TEM

**Fig. 6:**
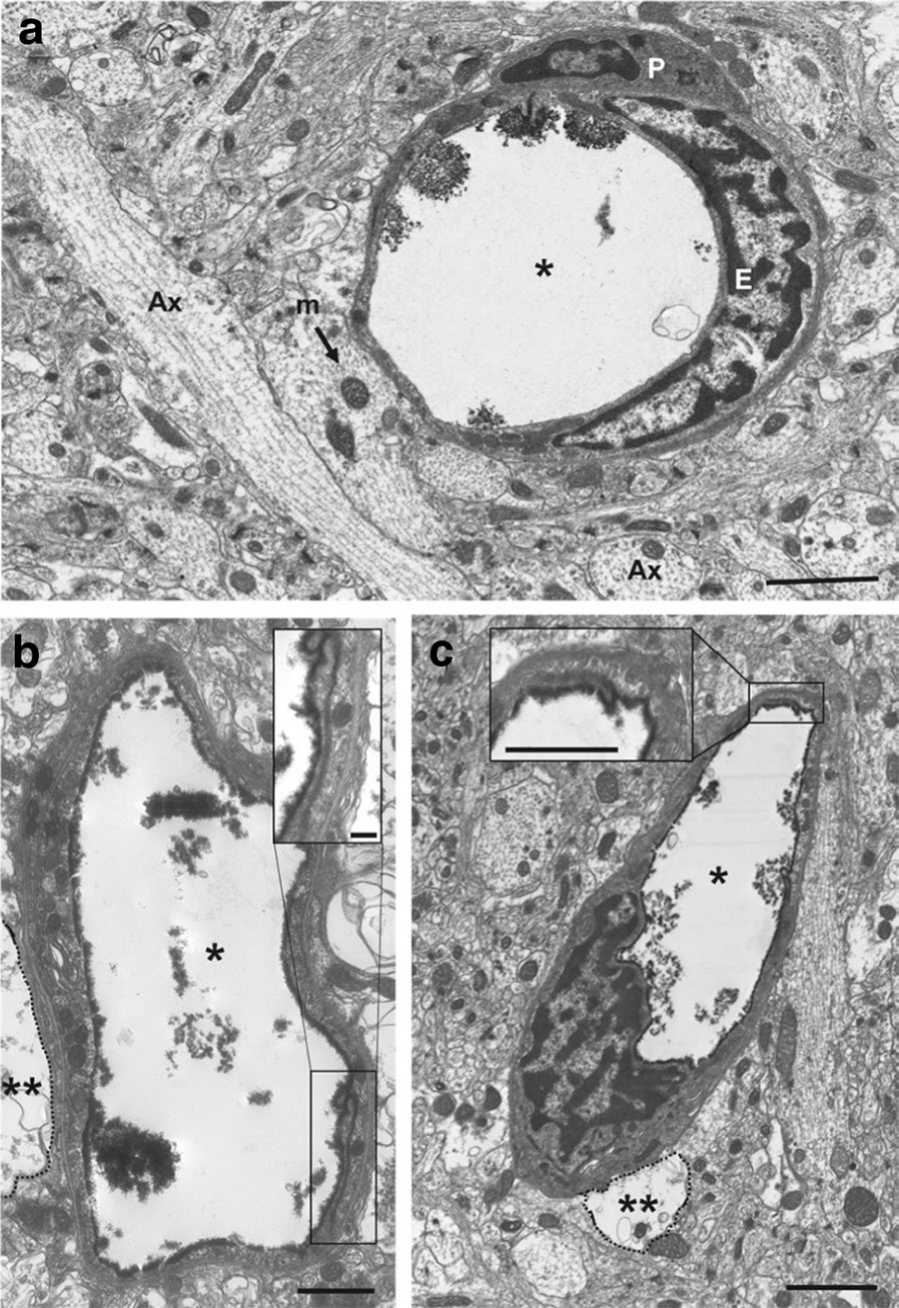
Electron micrographs of rat hippocampal capillaries. **a** Control showing the electron-opaque extracellular tracer (lanthanum nitrate) confined to the capillary lumen. **b** Capillary 1 h and **c** 3 h following rGO administration. Note that lanthanum nitrate lining the luminal inner surface is leaking through the inter-endothelial contact (*black boxes*). Insets show higher magnification of the contacts. *Ax* axon, *E* endothelial cell, *m* mitochondria, *P* pericyte; *asterisk*: lumen; *double asterisk, Dashed line* delineates regions of cytotoxic edema in perivascular astrocytes. *Bars* 2 μm (**a**, **c**), 1 μm (**b**, inset **c**), 200 nm (inset **b**)

TEM was used to assess whether hippocampal capillaries were permeable to the infusion of an electron dense extracellular tracer (lanthanum nitrate) after systemic administration of rGO. Figure 6 shows capillaries in the hippocampal parenchyma of control and rGO-treated rats that were systemically perfused with fixative containing the electron opaque extracellular tracer. In control rats, lanthanum nitrate was confined to the capillary lumen and the surrounding neuropile showed mitochondria, unmyelinated axons and synaptic contacts apparently normal-looking (Fig. 6a). In contrast, in capillaries of treated rats the extracellular tracer infiltrated the endothelial cleft, indicating a decrease in the paracellular tightness of the barrier after exposure to rGO (Fig. 6b; insets show details of the interendothelial cleft filled with tracer). This feature was more frequent at 3 h and was not observed at 7 days after rGO administration. The perivascular end-feet processes of astrocytes encircling leaked capillaries appeared swollen and were suggestive of cytotoxic edema. This was yet another indication that the BBB permeability was altered by the i.v. peripheral injection of rGO [33]. However, despite these changes, no extracellular tracer was observed filling the basement membrane or spreading through the neuropil interstitium. This finding suggested that rGO-induced alterations in the BBB did not cause vasogenic edema but simply induced abnormal astrocyte osmoregulation [34].

Detailed ultrastructural observations showed that leakage of the paracellular barrier was regionally highly heterogeneous since in some capillaries of rGO-treated rats the lanthanum nitrate was confined to the lumen, as in the controls. This finding suggested that the tightness of the BBB varied throughout the hippocampal parenchyma; regional heterogeneity in the permeability of the brain vasculature and tightness of the BBB, even in the same vascular segment, is not uncommon and has been well-documented [35].

### Molecular evaluation level by WB of hippocampal BBB paracellular pathway proteins

**Fig. 7:**
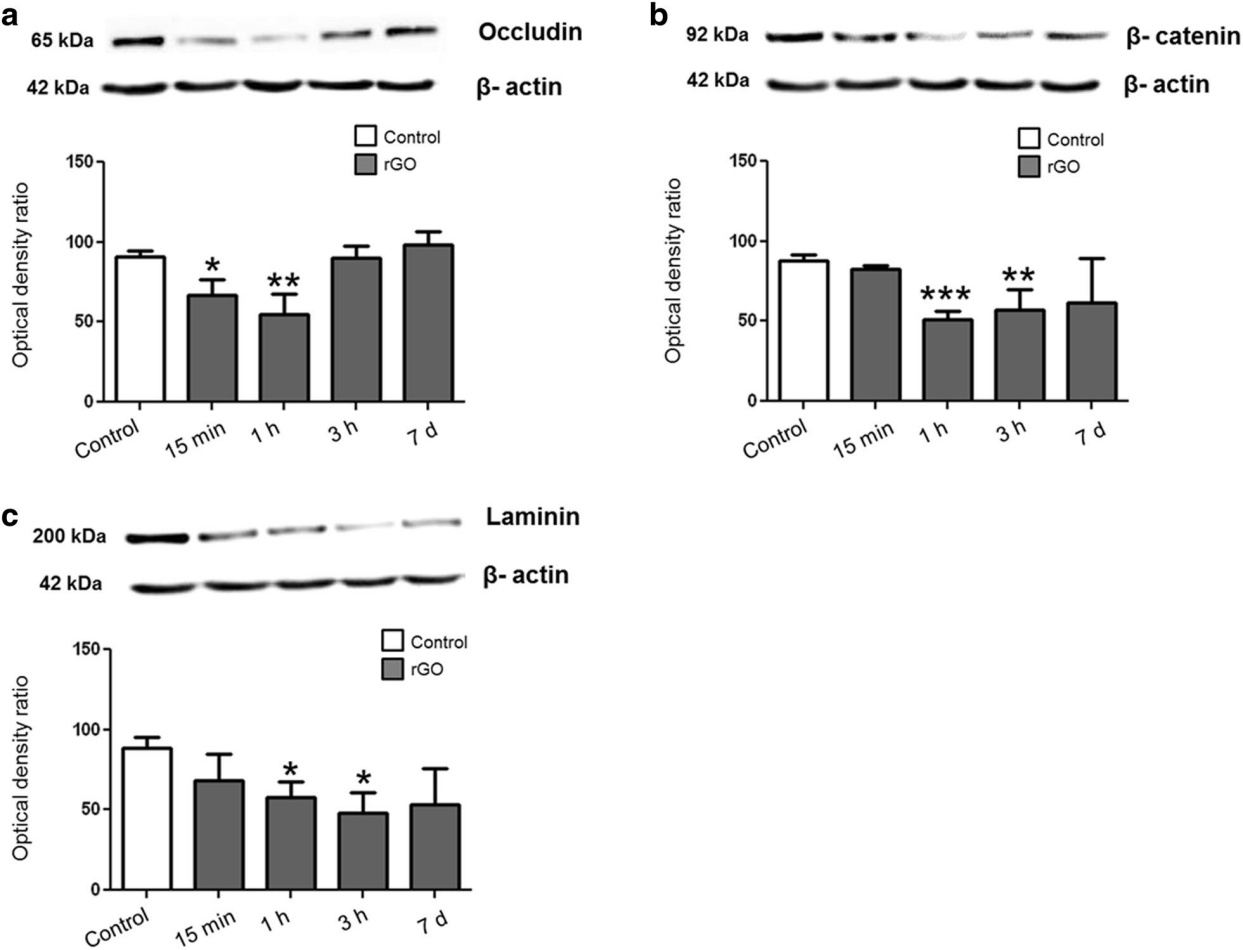
Molecular evaluation of hippocampal BBB paracellular pathway proteins as assessed by Western blotting. The panels show the expression of **a** occludin, **b** β-catenin and **c** laminin at different intervals after rGO administration (7 mg/kg, i.v.). Immunoreactive bands were quantified densitometrically and normalized to an internal standard (β-actin). The *columns* are the mean ± SEM (n = 5 rats/interval). *p < 0.05, **p < 0.01 and ***p < 0.001 compared to the corresponding control (Student’s *t*-test)

The western blotting analysis showing that the proteins associated with the impermeability of the BBB were down-regulated corroborated the TEM findings showing the extravasation of extracellular tracer at 1 h and 3 h post-rGO injection (Fig. 7). The expression of tight junction protein occludin, adherens junction protein β-catenin, and laminin from basal lamina was down-regulated at the same periods after rGO injection as shown by TEM. Occludin was the first protein to be affected, with significant downregulation (36 %) 15 min after rGO administration that reached 66 % after 1 h. β-catenin and laminin were decreased by 72 and 51 % at 1 h and 54 and 84 %, at 3 h, respectively. The immediate decrease in the levels of occludin and the ensuing decrease of laminin and β-catenin indicated that the systemic administration of rGO was able to disturb the paracellular permeability of the BBB. By day 7, the expression of all proteins had returned to control levels (Fig. 7a–c). These findings agreed with the progressive entry of rGO (15 min to 3 h) into the brain and their tendency to decrease to baseline (7 days) seen with MALDI-MSI. The short-lived disruption seen here indicated the reversible nature of the interference in BBB permeability caused by the rGO and confirmed a protective role for the BBB in normal brain function. In addition, the persistence of rGO within the brain for at least 1 week (as measured by MALDI-MSI) represents a good prospect for therapeutic strategies aimed at the long-term delivery of drugs [36] while at the same time reducing possible side-effects. Based on the findings of this study, we suggest that the use of rGO could be advantageous relative to other non-invasive approaches that disrupt the BBB, such as the use of ultrasound [37], hyperosmolar osmotic solutions (mannitol) [38] and pharmacological agents (histamine and bradykinin) [39] because of their ability to simultaneously modulate BBB permeability and act as drug delivery promoters.

## Conclusions

**Fig. 8:**
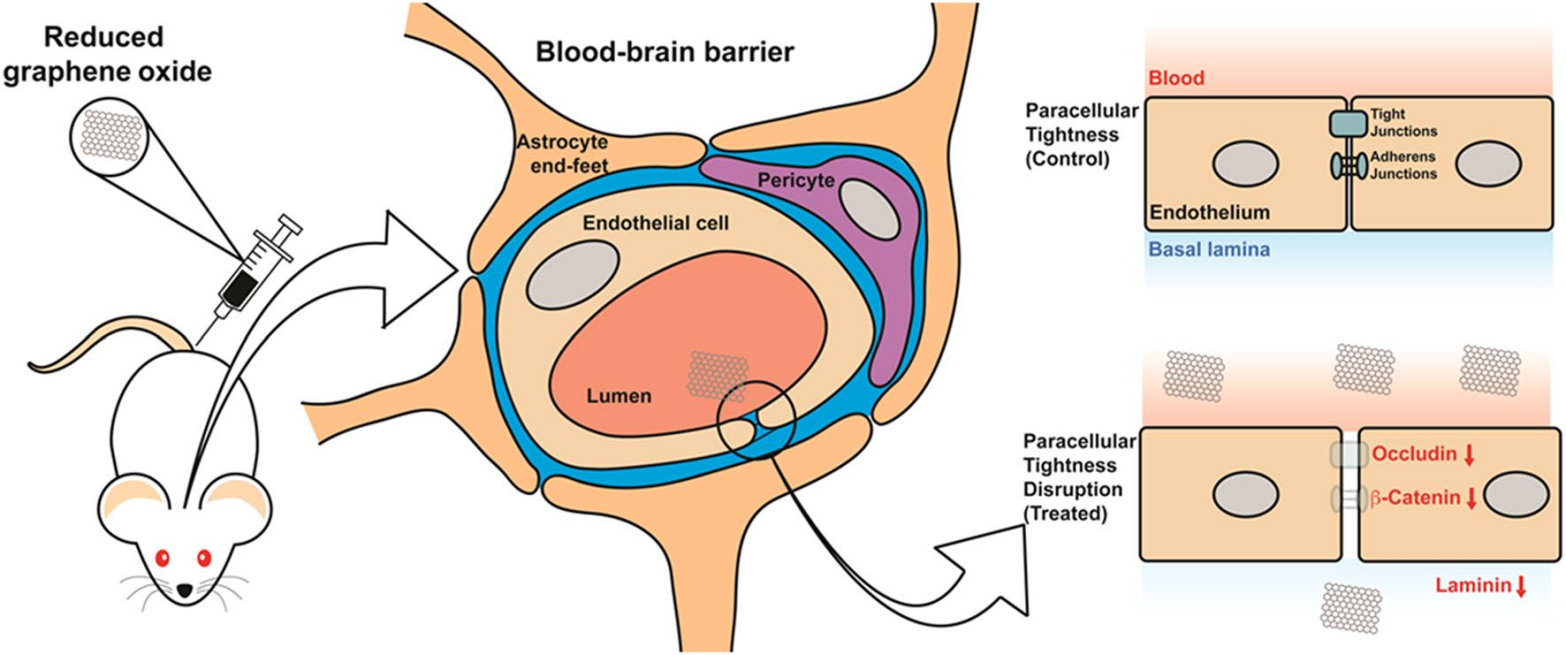
Schematic representation of the present experimental design aimed at demonstrating nanomaterial-based opening of the blood–brain barrier

We have characterized rGO produced in our laboratory and have described a novel application for MALDI-MSI in detecting the possible presence and temporal distribution of these particles in the brain after intravenous administration in rats. Although the crossing of rGO at the BBB was not shown, multiple resolution scale assessment revealed that in rGO-treated rats the BBB was time-dependently permeabilized through the weakening of the paracellular pathway. The transitory BBB breakdown after rGO injection was demonstrated by the passage of Evans blue dye from peripheral circulation to the brain, by lanthanum nitrate infiltration into the inter-endothelial cleft and by down-regulation of tight junction, adhesion junction and basement membrane proteins. Together, these findings indicate that administration of rGO decreases the paracellular tightness of the BBB (Fig. 8).

Although we still have to assess the toxicological effects of rGO, the results presented here suggest that rGO could be useful in providing nanomaterial-based therapy. Mainly, this could be potentially useful tool for treating a variety of brain disorders that are normally unresponsive to conventional treatment because of BBB impermeability.

## Methods

### rGO preparation and characterization

rGO was produced from catalytic conversion using hot-filament chemical vapor deposition process. Briefly, titanium substrate was immersed in 1 ml of polyaniline diluted in 2 ml of N-dimethylformamide, after which it was allowed drying for 2 h at room temperature. Then, 0.2 ml of nickel nitrate dissolved in 1 ml of pure acetone was added to the preparation. In the chemical vapor deposition chamber, tungsten filaments were heated (1500 °C) to decompose the reactive gases and heat the substrate. The hydrocarbons used as a carbon source were acetone, camphor and citric acid (1:1:1), which were dragged by hydrogen flow. The catalytic conversion is conducted under 10 Torr pressure and 65 sccm of nitrogen and 20 sccm of oxygen. The growth temperature (450 °C) was kept for 1 min in only hydrogen atmosphere in order to reduce the graphene oxide. After reduction, rGO was suspended in sterile distilled water (1 mg/ml) and sonicated for 5 min immediately prior to its use in experiments.

Morphological analysis of the sample was done with HRTEM. A drop of rGO aqueous suspension was deposited on carbon-coated copper grid. The excess water was removed by a filter paper; then, the samples were left to dry under ambient air and analyzed using a JEOL JSM-6330F microscope operated at 300 kV. The molecular structure was characterized by inVia Raman microscope (Renishaw, Wotton-under-Edge, UK) using the 514 nm wavelength of an argon-ion laser. All samples were deposited onto glass slides in powdered form without using vehicle. Absorption spectrum (800–200 nm) of rGO (70 µg/ml) was recorded at room temperature using a Varian Cary 5. rGO size distribution was measured by light scattering using a ZetaPALS Zeta Potential Analyzer (Brookhaven Instruments, NY, USA) at standard settings, typically 10 sub-runs of 30 s. Particle size and PDI were estimated using built-in software. The zeta potential was subsequently measured in the same sample using a conditioned electrode and was typically based on 10 runs in which the relative residual from model fitting was used. Each sample was measured three times consecutively.

### Animal care and rGO systemic administration

Male Wistar rats (*Rattus norvegicus*, 6 week-old, 180 ± 40 g) obtained from the Multidisciplinary Center for Biological Investigation at the State University of Campinas were housed 5/cage at 23 °C on a 12 h light/dark cycle, with lights on at 6 a.m. and access to water and food ad libitum. The animal experiments were approved by the institutional Committee for Ethics in Animal Use (protocol no. 2884-1) and were done in accordance with the ethical guidelines of the Brazilian Society of Laboratory Animal Science.

For toxicological assessment of nanomaterials involving intravenous (i.v.) administration, the stock solution concentrations of water-dispersible carbon nanostructures are generally ≤10 mg/ml and the maximum permissible dose is <10 mg/kg [40, 41]. Based on these guidelines, rats were injected via tail vein with a rGO suspension (7 mg/kg; 1.0–1.5 ml) or vehicle (sterile distilled water, control group) and, after 15 min, 1 and 3 h and 7 days, the rats (n = 3 per time interval for MALDI-MSI/confocal laser microscopy; n = 3 per time interval for Evans blue; n = 3 per time interval for TEM and n = 5 per time interval for WB) were killed with an overdose of a mixture (3:1) of ketamine chloride (Dopalen^®^, 100 mg/kg body weight) and xylazine chloride (Anasedan^®^, 10 mg/kg body weight) (Vetbrands, Jacarei, SP, Brazil) or CO_2_. A single control group killed 1 h after vehicle injection was used for comparison (n = 14) since preliminary experiments showed no time-related differences in the responses of control rats.

### Detection of rGO in the brain

#### MALDI-MSI analysis

At the time intervals indicated above, rats were anesthetized with an intraperitoneal injection of a mixture of ketamine chloride and xylazine chloride and perfused via the left ventricle with physiological saline followed by 4 % paraformaldehyde (PFA) in 0.1 M phosphate buffered saline (PBS), pH 7.4. The brains were quickly removed, post-fixed for 2 h in the same fixative and afterward successively cryo-protected in 15 and 30 % sucrose solutions (24 h each) at 4 °C. Since brain tissue is very friable, the samples were embedded in OCT-Tissue Tek (Sakura Finetek, Torrance, CA, USA) and frozen in n-hexane (Dinâmica, São Paulo, SP, Brazil), cooled with liquid nitrogen and stored at −80 °C until cryo-sectioning (Leica CM 1850 Cryostat; Milton Keynes, UK). Cryo-sections 10 μm thick were collected on glass microscope slides that were then covered with a matrix solution (synapic acid, 10 mg/ml in 60:40 acetonitrile:H_2_O) using a commercial airbrush. Images and mass spectra were acquired in a MALDI-LTQ-XL MSI instrument (Thermo Scientific, Carlsbad, CA, USA) equipped with a laser (Nd:YAG, 355 nm) at *minimum* focus setting and a quadrupole-ion-trap analyzing system in negative ion mode. For image acquisition, a 100 µm raster width was selected. The data were standardized using collision-induced energy to 35 eV. Helium was used as the collision gas. All imaging data were processed using ImageQuest software v.1.0.1 (Thermo Scientific).

Mass and intensity values for each spectrum were included in the Principal Component Analysis (PCA), which was performed using Unscrambler v.9.7 (CAMO Software, Trondheim, Norway). After discrimination by PCA, MS/MS reactions were performed to generate their fragmentation pattern.

**Fig. 9:**
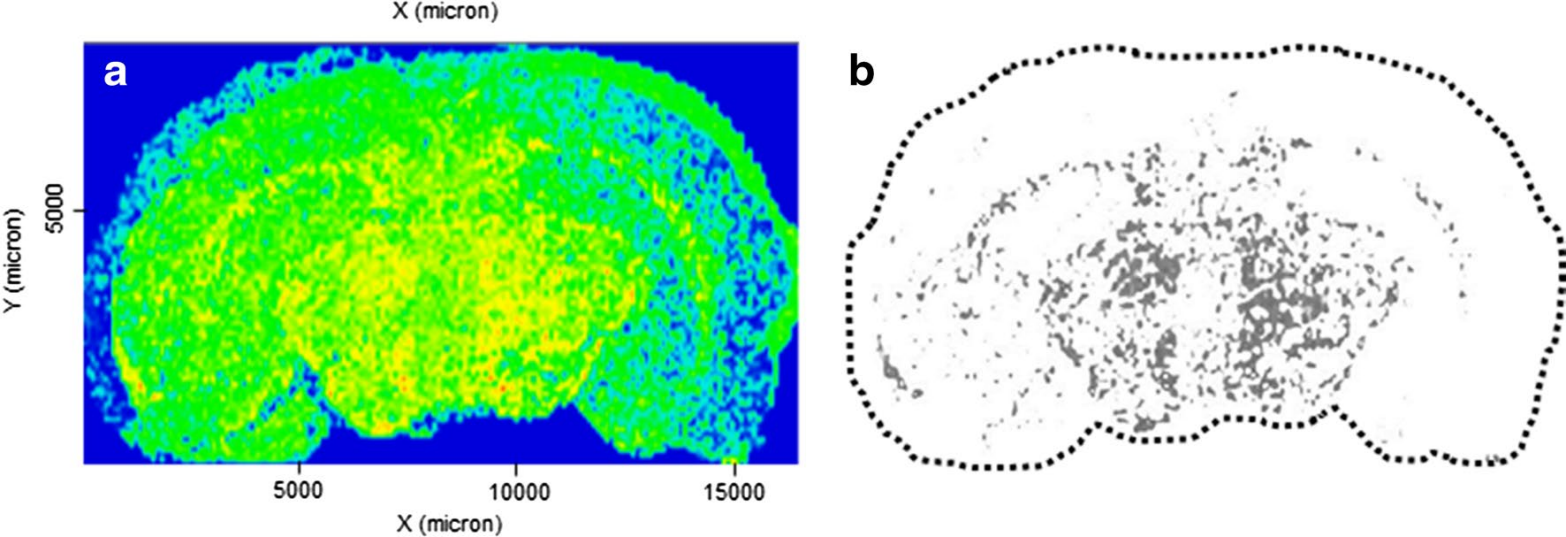
Methodology used for quantitative analysis of rGO. **a** Representative MALDI-mass spectrometry imaging of rGO-treated brain section (Fig. 3c). **b** The same section after color segmentation using GIMP 2.8 software

The quantification and distribution of rGO in the brain were assessed using GNU Image Manipulation Program (GIMP) 2.8 software that converted the digitized MALDI images to grayscale images (n = 3 images/time, i.e., one image per rat; n = 3 rats/time) after yellow color-based selection [42]; in each image, the range of yellows shading the dots, and representing the rGO depth into tissue, were selected dynamically by the program which thus converted them to digitized grayscale dots and further to pixels’ intensity. The mean of the sum of pixels in each time point was plotted as histogram. The red dots were not computed in terms of pixels given its paucity and minimal distribution. Figure 9 illustrates the appearance of spatial distribution of yellow dots representing rGO nanoparticles following computer-based image conversion to digitized grayscale.

### Confocal laser microscopy analysis

The same samples prepared for MALDI-MSI analysis were examined using a Zeiss LSM 780-NLO confocal imager fitted on an Axio Observer Z.1 microscope (Carl Zeiss AG, Germany) equipped with a 60× oil immersion lens. The images were acquired using the pinholes set to 1 airy unit for each channel in a sequential manner and analyzed using ImageJ 1.45 s software (NIH, Bethesda, USA). The images of rGO were obtained using two-photon excitation with 780-nm laser pulses and emission spectra were recorded between 611 and 735 nm [43, 44].

### Evaluation of BBB integrity

#### Gross analysis by Evans blue peripheral infusion

BBB integrity was first examined by assessing Evans blue dye extravasation [45] Briefly, 10 min prior the end of each time course, Evans blue dye solution was injected (10 mg/kg of 2 % dye in 0.9 % NaCl) intravenously through the tail vein. Animals were killed with an overdose of anesthetic and the brains were rapidly removed and photographed.

#### Ultrastructural analysis of extracellular tracer leakage

One hour after vehicle administration and at various intervals after rGO injection, rats were anesthetized with ketamine/xylazine and killed by transcardial perfusion with 80 ml of 0.9 % NaCl followed by 250 ml of fixative solution (2.5 % PFA, 2.5 % glutaraldehyde plus 2 % lanthanum nitrate in 0.1 M sodium cacodylate, pH 7.2) as described in previous study [46, 47]. The rats were maintained at 4 °C for 12–18 h to allow adequate fixation before brain dissection and processing in order to minimize tissue artifacts. The brain was then excised and the hippocampi were dissected. Hippocampi were selected for analysis because this was a brain region in which an appreciable concentration of rGO was found by MALDI-MSI. Tissue samples 0.5–1 mm thick were immersed in the same fixative (lanthanum nitrate-free) for 2 h. After rinsing, the samples were post-fixed in 1 % osmium tetroxide plus 1 % potassium ferricyanide in glucose solution (0.2 M sucrose plus 0.1 M NaCl) for 1 h, washed three times and dehydrated in a graded ethanol series (50–100 %), ethanol:acetone (1:1) and 100 % acetone, followed by infiltration with acetone:Epon (1:1) and embedding in Epon 812 (Sigma Aldrich, St. Louis, MO, USA) resin for 24 h at room temperature. The samples were subsequently transferred to fresh resin for polymerization (72 h at 60 °C). The areas of interest were chosen by histological analysis of semi-thin sections (0.5 μm thick) stained with 0.5 % toluidine blue. Ultrathin (70 nm thick) sections from selected areas were placed on 200-mesh copper grids, double contrasted with 2 % uranyl acetate (in 70 % methanol) followed by 0.5 % lead citrate aqueous solution before examination in a Zeiss LEO 906 transmission electron microscope (Carl Zeiss MicroImaging GmbH, Göttingen, Germany) operated at 60 kV.

#### Western blotting of proteins associated with the BBB in hippocampal homogenates

At 15 min, 1 h, 3 h and 7 days after rGO i.v. injection or 1 h after vehicle injection, the rats were anesthetized by CO_2_ inhalation and killed by decapitation. Protein was extracted from hippocampus as previously described [48]. After electrotransfer, the membranes were incubated with 5 % skimmed milk to block non-specific sites prior followed by washing with TBS-T (0.1 % Tris-buffered saline with 0.05 % Tween 20, pH 7.4). Subsequently, the membranes were incubated with primary antibodies against β-catenin (1:500; sc-7963, Santa Cruz Biotechnology, Santa Cruz, CA, USA), occludin (1:500; sc-5562, Santa Cruz) and laminin (1:500; L9393, Sigma Aldrich) followed by washing with TBS-T and incubation with HRP-labeled anti-mouse (for anti-β-catenin and anti-β-actin) or anti-rabbit (anti-occludin and anti-laminin) secondary antibody (1:1000, Sigma Aldrich). Immunoreactive bands were visualized using a chemiluminescence kit (Super Signal West Pico Chemiluminescent Substrate; Pierce Biotechnology, Rockford, IL, USA). The blots were subsequently stripped and probed with anti-β-actin (1:1000; A2228, Sigma Aldrich); β-actin was used as an internal control to monitor protein loading, the efficiency of blot transfer, and nonspecific changes in protein levels. The luminescent signal of each band was captured with a G:BoxiChemi camera (Syngene, Cambridge, UK) and band intensity was quantified using ImageJ 1.45 s software (NIH, Bethesda, MD, USA).

### Statistical analysis

Quantitative data were expressed as the mean ± standard error of the mean (SEM). Statistical significance was determined by one-way ANOVA followed by the Bonferroni post hoc test for multiple variant analysis and Student’s *t* test for pairwise analyses. A value of p < 0.05 indicated significance. All analyses were done using Prism software, version 5 (GraphPad Inc., La Jolla, CA, USA).

## Abbreviations

AJ: adherens junction
ANOVA: analysis of variance
BBB: blood–brain barrier
HRTEM: high-resolution transmission electron microscopy
MALDI-MSI: matrix-assisted laser desorption/ionization mass spectrometry imaging
PBS: phosphate buffered saline
PCA: Principal Component Analysis
PDI: polydispersity index
PFA: paraformaldehyde
rGO: reduced graphene oxide
SEM: standard error of the mean
TEM: transmission electron microscopy
TJ: thigh junction
WB: western blotting
